# Dental care resistance prevention and antibiotic prescribing modification—the cluster-randomised controlled DREAM trial

**DOI:** 10.1186/1748-5908-9-27

**Published:** 2014-02-22

**Authors:** Christin Löffler, Femke Böhmer, Anne Hornung, Hermann Lang, Ulrike Burmeister, Andreas Podbielski, Anja Wollny, Günther Kundt, Attila Altiner

**Affiliations:** 1 Institute of General Practice, Rostock University Medical Center, Postfach 100888, 18055 Rostock, Germany; 2 Department of Conservative Dentistry and Periodontology, Rostock University Medical Center, Rostock, Germany; 3 Institute of Medical Microbiology, Virology and Hygiene, Rostock University Medical Center, Rostock, Germany; 4 Institute of Biostatistics and Informatics in Medicine and Ageing Research, Rostock University Medical Center, Rostock, Germany

**Keywords:** Dentistry, Public health dentistry, Dental care for chronically ill, Anti-bacterial agents, Antibiotic prophylaxis, Health communication, Drug resistance

## Abstract

**Background:**

Bacterial resistance development is one of the most urgent problems in healthcare worldwide. In Europe, dentistry accounts for a comparatively high amount of antibiotic prescriptions. In light of increasing levels of bacterial resistance, this development is alarming. So far, very few interventional studies have been performed, and further research is urgently needed. By means of a complex educational intervention, the DREAM trial aims at optimising antibiotic prescribing behaviour of general dentists in Germany.

**Method:**

This is a cluster-randomised controlled trial, where each cluster consists of one dental practice and all of its patients in a defined period. Participants are general dentists practicing in the German region of Mecklenburg-Western Pomerania. Randomisation takes place after baseline data collection (6 months) and will be stratified by the antibiotic prescribing rates of the participating dental practices. Dentists randomised into the intervention group will participate in a complex small group educational seminar that aims at: increasing knowledge on bacterial resistance, pharmacology, and prophylaxis of infectious endocarditis; increasing awareness of dentist-patient communication using video-taped vignettes of dentist-patient communication on antibiotic treatment; improving collaboration between general dentists, general practitioners, and practice-based cardiologists on the necessity of antibiotic prophylaxis; enhancing awareness of the dentists’ own prescribing habits by providing antibiotic prescribing feedback; and increasing patient knowledge on antibiotic treatment by providing patient-centred information material on antibiotic prophylaxis of endocarditis. The dentists randomised into the control group will not receive any educational programme and provide care as usual. Primary outcome is the overall antibiotic prescribing rate measured at T1 (period of six months after intervention). In a subgroup of adult patients affected by odontogenic infections, microbiological analyses for antibiotic resistance of oral streptococci are performed.

**Discussion:**

Major aim of the study is to improve the process of decision making with regard to antibiotic prescribing. The approach is simple to implement and might be used rapidly in graduate and post-graduate medical education. We expect the results of this trial to have a major impact on antibiotic prescription strategies and practices in Germany.

**Trial registration:**

Current Controlled Trials ISRCTN09576376

## Background

Rising levels of bacterial resistance are one of the most urgent problems in healthcare worldwide. The overuse and the misuse of antibiotics are the basic reasons for this development. Studies consistently confirm the association between antimicrobial consumption and bacterial resistance [1]. As a consequence, in order to reduce and slow down the global threat of bacterial resistance development, it is very important to reduce the inappropriate use of antibiotics. Antimicrobial drugs should be reserved to patients who actually benefit from the treatment.

Apart from this severe global development, at the individual level, treatment with antibiotics correlates with drug-related adverse reactions, such as diarrhea or allergic reactions. Especially, if antibiotics are not indicated, these reactions outweigh any benefit of the treatment [2].

Prescribing non-indicated antibiotics also has an impact on healthcare expenditures. Both unnecessary consumption of antibiotics and treatment of preventable drug-related adverse reactions, including hospital stays, put a huge financial burden on national healthcare systems. Optimising the use of antibiotics will decrease this burden [3].

Data on outpatient antibiotic use show that since 1997 daily defined doses per 1,000 inhabitants per day (DID) have increased in Europe. At the same time, prescriptions of broad spectrum antibiotics have become more common, while narrow spectrum antibiotics have been used less [4,5]. This development is fostering further bacterial resistance. With 7% of all antibiotics used in primary care, dentistry accounts for a comparatively high amount of antibiotic prescriptions [6]. Studies indicate a non-conforming use of antibiotics as well as a suboptimal choice of antimicrobial substances as far as guideline recommendations are concerned [7,8]. In light of increasing levels of resistance, this development is alarming [6].

As to general dentistry in Germany, little data exists. However, comparing absolute numbers of antibiotic prescriptions in primary care, dentists are fourth, following general practitioners, internal specialists, and pediatricians [9]. Data of German statutory health insurances on the year 2010 show that, on average, dentists prescribed two antibiotics per week. Interestingly, in derogation from national and international guidelines, German general dentists seem to favor clindamycin over preferably recommended substances [10]. A study performed in northern Germany and based on postal questionnaires shows similar results [11].

Optimising antibiotic prescribing in general dentistry might contribute to decreasing levels of antibiotic resistance development and thereby increase the quality of healthcare provision in general. In the past, several interventional randomised controlled trials (RCTs) from other medical fields were successful in optimising antibiotic prescribing. Most of these studies focused on respiratory tract infections, the disease that causes most antibiotic misuse and overuse in primary care. Results show that complex interventions that aim at changing behaviour rather than just providing information are most efficient [1,12-14].

Concerning dentistry, very few interventional studies have been performed so far. Palmer *et al.*, for example, collected data of antibiotic prescribing among general dentists six weeks before and after an audit that included educational components and the provision of guidelines. Antibiotic prescriptions decreased by 42.5%. Above that, guideline conformity could be improved [15]. Despite the very promising results, the study lacked a long-term follow-up. In a three-armed RCT performed by Seager *et al.*, the efficiency of educational outreach visits was compared to the provision of information material by mail in an intervention group and to no intervention in a control group. The educational outreach visits were successful in reducing prescribing rates significantly and also improved prescribing appropriateness [16]. In the field of dentistry, where a high potential for reducing non-indicated antibiotics exists, further research is urgently needed.

To investigate the underlying reasons for inadequate antibiotic treatment in German general dentistry, within the DREAM trial we performed extensive qualitative research. Narrative in-depth interviews with dentists revealed areas and situations of non-indicated antibiotic prescribing. These include treatment of patients during emergency services or shortly before weekends or holidays when, due to a lack of time and/or follow-up, dentists were inclined to prescribe antibiotics. Moreover, the treatment of patients suffering from multimorbidity was perceived as a challenge. Both the lack of medical knowledge on cardiac impairments and the fear of judicial consequences induced dentists to prescribe antibiotics for safety reasons. Based on these results, approaches to solve these challenging situations were developed. Especially with regard to their acceptability and practicability, these approaches were discussed extensively among dentists by means of focus group discussions. The results contributed significantly to the development of the complex intervention.

### Objectives

By means of a complex educational intervention, the cluster-randomised controlled DREAM trial aims at optimising antibiotic-prescribing behaviour of general dentists in Germany. The complex educational intervention is based on narrative in-depth interviews and focus group discussions with dentists and includes transfer of relevant knowledge, dentist-patient communication, improved collaboration with physicians from other fields, antibiotic prescribing feedback, and patient education. The trial will test the efficiency of the intervention. In addition, the study contains a subset of microbiological analyses on antibiotic resistance among a subgroup of patients affected by odontogenic infections.

## Methods

### Trial design

A cluster-randomised controlled trial (cRCT) will be performed. Clusters are employed to avoid contamination between the intervention and the control group. Each cluster will consist of one general dentist practice and all of its patients during a defined period. Prior to randomisation, baseline data will be collected over a period of six months (T0) to adjust for inter-cluster imbalances of antibiotic prescribing. After randomisation, the intervention takes place which is followed by two more six-month periods of collecting antibiotic prescribing data (T1 and T2).

### Participants

General dentists with their practices in the German region of Mecklenburg-Western Pomerania are eligible for participation in the trial. Given their highly selective group of patients, dentists with sub-specializations will not be included. Among the participating dentists, antibiotic prescribing data of all patients will be collected.

### Recruitment

All general dentists with a practice in and around 150 kilometers of Rostock (n = 665), the largest city of Mecklenburg-Western Pomerania, will receive postal invitations to take part in the study. These invitations will be sent out in waves and will be based on a random sample of eligible dentists. Participating dentists will receive case payments to compensate for time and effort. The payments are identical in the intervention and the control group. Trained study assistants take care of the enrolment process. Participating dentists provide informed consent before baseline data collection and randomization.

### Randomisation

Cluster-randomisation takes place after baseline data collection. Concealment of allocation is preserved. Randomisation will be stratified by the antibiotic prescribing rates of the participating dental practices and will be performed by a statistician; this will avoid imbalances between the intervention and the control group.

### Blinding

Within the DREAM trial, it will not be possible to blind dentists, study personnel, and statisticians.

### Intervention

After randomisation, dentists of the intervention group will participate in a four-hour, small-group intervention seminar. The complex educational seminar is based on a review of the literature and on own extensive qualitative research. The seminar addresses the most challenging situations in the decision-making relating to antibiotic prescribing in general dentistry. It includes condensed transfer of relevant knowledge on bacterial resistance, pharmacology, and prophylaxis of infectious endocarditis; in-depth discussion on dentist-patient communication using three video-taped examples of poor, good, and elaborated dentist-patient communication on antibiotic treatment—the discussion particularly aims at elaborating strategies to satisfy dentists’ and patients’ needs for safety without prescribing non-indicated antibiotics; improved collaboration between dentists, general practitioners, and practice-based cardiologists by means of a standard fax form to agree upon the need for an antibiotic prophylaxis of endocarditis; antibiotic prescribing feedback that will be provided every six months, and will enhance dentists’ awareness of their own prescribing habits compared to those of their colleagues; and patient-centred information material to be used in the dentists’ practices. The information on the necessity to use or not use an antibiotic prophylaxis of endocarditis shall increase patients’ knowledge on antibiotic treatment.

### Control group

During the trial, the dentists of the control group receive neither the seminar nor the information material. They will provide care as usual. After the trial, the dentists of the control group will be invited to take part in the seminar.

### Outcomes

Primary outcome is the overall antibiotic prescribing rate of the intervention group and the control group measured at T1, thus measured in the interval between months one to six after intervention. A number of secondary outcomes will be analyzed for the subgroup of patients suffering from odontogenic infections (see below).

### Sample size

**Figure 1:**
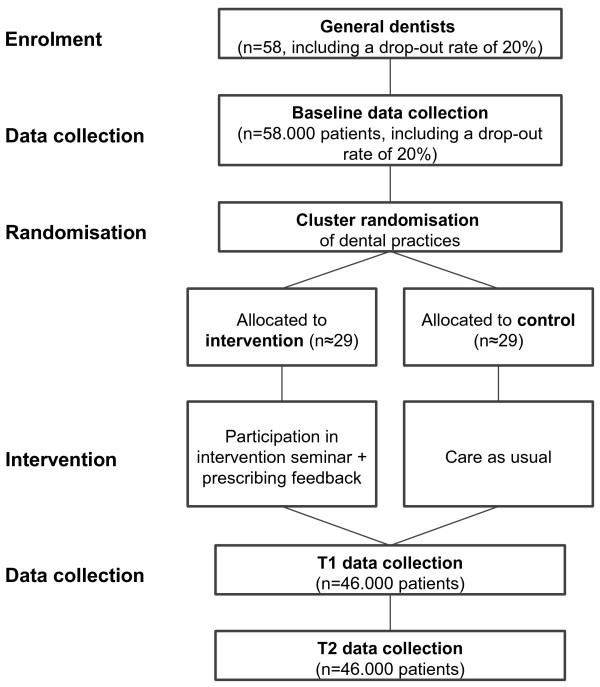
Flow chart of the DREAM trial.

Data from Northern Germany shows that about 6% of all patients cared for in general dentistry receive antibiotics [11]. A relative reduction of 20% is perceived as both clinically relevant and attainable. For comparison, the previous CHANGE cRCT on antibiotic prescribing in German primary care reached a relative reduction of 40% [14]. The estimated intra-class correlation coefficient (ICC) of the prior CHANGE trial was employed for sample size calculation in this trial. In a RCT randomising at patient level and aiming at a reduction of antibiotic prescribing from 6% to 4.8% (a relative reduction of 20%) about 5,750 patients per group are necessary (power = 80%, level of significance (two-sided) α = 5%). Using an ICC of 0.2 and a cluster size of 1,000 patients the sample size needs to be adjusted with an average design factor of 4. Thus a total sample size of 46,000 patients in 46 dental practices is necessary for the trial. Assuming a dropout rate of 20% initially, 58 practices need to be recruited for baseline (see Figure 1).

### Study on antibiotic resistance of oral streptococci

In a subgroup of adult patients suffering from odontogenic infections, microbiological analyses on antibiotic resistance of oral streptococci are conducted. Intraoral swabs from the buccal mucosa will be collected of these study patients at three points in time: at the first consultation due to an odontogenic infection (T0), after two weeks (T1), and after six months (T2). The swabs will be transported at room temperature to the accredited diagnostic laboratory within two hours. There, the swabs will be streaked onto sheep blood agar plates. After 48 hours of incubation under a CO2-enriched atmosphere, samples from every morphologic type of alpha-hemolytic colonies will be gained for species identification and for determination of the antibiotic resistance profile. For each patient, the temporal changes in present oral streptococcal species and antibiotic resistance profiles will be recorded.

In the context of the overall cRCT, objective information about antibiotic resistance development will be correlated to antibiotic prescribing behaviour of dental care practices. This information will be communicated to dentists (via feedback) to enhance positive competition. For this subgroup, we intend to include a total number of 500 patients suffering from odontogenic infections. Among these patients, several secondary outcomes will be assessed. These include severity of the infection (judged by the dentist), pain severity, duration of therapy, re-consultation rate, adverse drug events (with reference to antibiotics), complications, and antibiotic resistance developments.

### Data collection, completeness, and quality

Data collection will be based on practice software. In Germany, dentists make use of a large variety of software packages. Trained study assistants will visit the participating practices regularly to collect data and to verify data quality by comparing antibiotic prescriptions and number of patient visits with software entries. After data collection, a second study assistant will check the data file for completeness and correctness.

### Statistical methods

A ‘full analysis set’ (FAS) following the principle of intent-to-treat (ITT) will include every patient as randomised. First, confirmatory analyses on efficacy variables will be performed on the FAS patients. Because imbalances are more likely among cluster-randomised trials than among trials randomised at the patient level, variables related to dentists’ characteristics will already be included in the primary data analysis. Initially, baseline characteristics will be compared between groups. If significant differences are found for variables that could potentially bias the results, data analysis will be based on a comparison of the baseline-adjusted rates of antibiotic prescribing between the intervention and the control group at T1 (primary outcome) and T2. The direct maximum likelihood approach is used as estimation procedure and will provide unbiased estimated values. Analyses for secondary endpoints will be performed in a strictly exploratory way using analogue models.

### Process evaluation

After T1 a short standardised questionnaire and some qualitative in-depth interviews with dentists of the intervention group will be performed. The interviews will focus on the usefulness and usability of the material offered during the intervention seminar (*e.g.*, standard fax form for dentist-physician communication or patient information material) and the way dentists made use of it. Recommendations for improvement will also be collected. The interviews will be recorded, transcribed, and analysed.

### Ethical approval

The study was approved by the Ethics Committee of the Rostock University Medical Center in December 2012 (Approval-No. A 2012-0147).

### Study registration

The study has been registered with Current Controlled Trials Ltd. with the reference ISRCTN09576376.

### Trial status

Currently (the end of 2013), the baseline data have been collected, the participating dentists have been randomised, and the dentists of the intervention group have been trained. The T1 data collection period has started. The data on T1 will be collected by the end of the T1 six-month period in spring 2014.

## Discussion

In light of rising levels of antibiotic resistance worldwide, reducing the number of non-indicated prescriptions of antibiotics is highly relevant. A substantial part of antibiotic resistance is caused in primary care [17,18]. This has an impact on secondary healthcare as well: the increasing antibiotic resistances in ambulatory care cause a growing use of broad-spectrum antibiotics in hospitals, a process that fosters further resistance development. Although several previous studies have aimed at reducing antibiotic prescribing in general practice and/or pediatrics [1,12-14], so far hardly any initiative has focused on dentistry. This is rather surprising, because dentistry accounts for a comparatively high amount of antibiotics and witnesses increasing resistance levels among dental pathogens. By means of a complex educational intervention, the DREAM trial takes up this fact and aims at optimising antibiotic prescribing behaviour of general dentists in Germany.

The modeling of the intervention is based on concepts, such as communication training or prescribing feedback, that have already proven their efficiency in reducing non-indicated antibiotic prescribing in general practice [13,14]. By performing narrative in-depth interviews and focus group discussions with dentists, these concepts have been adapted to the specific setting of dentistry. Dentist-patient communication, for example, is subject to specific constraints: During treatment, patients usually have few possibilities to talk to their dentist. As a consequence, gestures and facial expressions as well as communication at the beginning and at the end of the consultation are especially important. The intervention seminar takes these specific conditions into account.

As to bacterial resistance development of oral streptococci, very little research exists. A study by Chardin *et al.* found that, in a healthy population, treatment with amoxicillin—which is commonly used in dentistry—increases antibiotic resistance [19]. The study on antibiotic resistance of oral streptococci performed in this trial will enhance evidence in this field. Results of these analyses will be communicated to dentists and—by means of a positive competition—are assumed to have a positive impact.

## Conclusion

In case of positive evaluation, the intervention seminar developed for the DREAM trial might serve as a theoretically grounded and efficient concept tested for feasibility and acceptability. Consequently, it might be integrated into graduate and post-graduate education. Because the intervention seminar is easy to implement and demands relatively little time from the dentists, it might be transferred to other healthcare systems. This way, the approach might contribute to a sustainable reduction of antibiotic prescribing in general dentistry.

## Abbreviations

DID: Daily defined doses per 1000 inhabitants per day; FAS: Full analysis set; ICC: Intra-class correlation coefficient; ITT: Intention-to-treat (analysis); RCT: Randomised controlled trials.

## Competing interests

The authors declare that they have no competing interests.

## Authors’ contributions

AA, HL, AP and CL initiated and designed the study; all authors performed further development. The paper was drafted by CL and FB and all authors read and approved the final manuscript.
